# Biological, cultural, and environmental factors catalyzing the emergence of (alternate) sign languages

**DOI:** 10.3389/fpsyg.2023.1224437

**Published:** 2023-10-19

**Authors:** Aritz Irurtzun

**Affiliations:** CNRS, IKER (UMR 5478), Bayonne, France

**Keywords:** alternate sign languages, language design, modality, deafness, speech taboo

## 1. Introduction

In the last decades, there has been a growing number of studies analyzing the extent to which and the mechanisms by which language-external factors affect particular aspects of the design of human language(s).

Here I want to make a plea for what I consider are the clearest and most spectacular cases of language-external factors variably affecting language design. I argue that the choice of modality of a language (spoken/gestural) can be independently determined by (i) biological, (ii) cultural, and (iii) environmental factors. What is more, these will not be factors affecting cumulative diachronic language change, but rather language design *ex nihilo* —to the extent that these are “new” languages, i.e., not derived by regular diachronic change of the local oral language structures^1^. Thus, they constitute evidence against any *a priori* skeptical view on the possibility for language-external factors to substantially affect core aspects of the grammar of languages (see e.g., Benítez-Burraco and Moran, 2018 for discussion).

## 2. Biological factors

The ethno-linguistic and anthropological literature has not yet attested any human population that in the absence of a widespread deafness does not resort to the oral-auditory channel (i.e., speech) for the externalization of language. It seems to be a strongly biased option. It does not matter whether the first human languages were gestural or vocal (*cf*. De Condillac, 1746; Hewes, 1973; Emmorey, 2005; Fay et al., 2014; Cooperrider, 2020), the observation is that in any human group where there is no particular prevalence of deafness, there is at least an oral language that is employed for intragroup communication. In other words, all things being equal, human populations employ languages that privilege the oral-auditory channel of externalization to the gestural-visual one even if often speech is accompanied by gesture (see, i.e., Kendon, 2004; Enfield, 2009).

However, if conditions such as congenital deafness are widespread in a community, languages privileging the gestural-visual channel tend to emerge^2^. This is famously the case of Martha's Vineyard Sign Language (Groce, 1985), of Al-Sayyid Bedouin Sign Language (Padden et al., 2010; Sandler et al., 2014), and of any other village sign language (see e.g., Zehsan and de Vos, 2012). A famous case of sign-language emergence concerns the Nicaraguan Sign Language (Kegl and Senghas, 1999), which emerged when deaf homesigners were gathered for the first time, creating thus a community that could interact and generate the primary linguistic data for new generations of deaf learners. Furthermore, in the absence of both hearing and sight, deafblind people employ different types of tactile sign languages to different degrees (Mesch, 2001, 2013; Dammeyer et al., 2015; Checchetto et al., 2018)^3^.

These are, I believe, clear and indisputable cases of language-external factors affecting language structure. And note that even if there are remarkable similarities between spoken and signed languages, modality seems to play a crucial role in determining certain aspects of language-design that go beyond phonology (say, the general use of classifiers, clause-final *wh*-phrases, inflectional paradigms, particularities of the spatial systems for deixis and reference, etc, *cf*. i.e., Swisher, 1988).

Nevertheless, biology is not the only factor variably affecting language design; cultural and even environmental factors too can modulate the choice of modality (and in consequence, of certain structural traits of languages), as we will see next.

## 3. Cultural factors

In this section I discuss a range of cultural factors that partake in the emergence and spread of alternate sign languages (sign languages employed by hearing individuals to communicate between them in particular occasions). These are related to speech taboos, to the valuing of silence in specific cultural niches, and to the communication impediment in language-clash situations.

### 3.1. The value of silence

Certain cultural norms can lead individuals being exposed to environments where they must privilege the gestural-visual channel to communicate in silence. Patently, this is the case of traditional hunting expeditions, where not being perceived (heard) by the prey is of utmost strategic value. Some human populations such as the San of Southern Africa have developed hand gesture communication systems for that end; linguistic systems that allow them to communicate while remaining unnoticeable to the prey (see i.e., Lewis, 2009; Mohr and Fehn, 2013; Hindley, 2014; Mohr, 2015, 2017; Sands et al., 2017; Mohr et al., 2019).

The case of the various Australian Aboriginal Sign Languages also fits this pattern. These languages (employed by over 80 different human groups—from the Arrernte to the Warramunga—, *cf*. Kendon, 1988) have been used on a daily basis to communicate in silence. As in the case of Southern Africa, this can serve the strategic goal of not being heard in hunting parties, but it can also obey to considerations of tact or social discretion, or serve in multi-disciplinary traditional storytelling (Green, 2014). Last, there is (or has been) a widespread speech-taboo imposed onto widows by which they have to remain silent for a variable mourning period in which case they have to resort to the sign language^4^. The logic under this speech taboo comes from the emic consideration that the soul of the deceased lingers in this world for a while before going to the world of the spirits, and thus, had he heard the voice of his widow, he may have stayed without accomplishing the passage^5^^,^^6^^,^^7^. Furthermore, the taboo also extends to other passage rituals given that “[n]ovices during initiation ceremonies are ritually dead. Dead people cannot speak, therefore novices on the ceremonial grounds should converse only in signs” (Meggitt, 1954, p. 4).

Another famous instance of sign-language emergence in a cultural niche highly valuing silence is the monastic sign languages (*cf*. Gougaud, 1929; Barakat, 1975; Umiker-Sebeok and Sebeok, 1987; De Saint-Loup et al., 1997; Bruce, 2007; Quay, 2015). This is a movement that started within the abbey of Cluny in Burgundy, where the doctrine was to advocate for an angelic behavior of its monks. The Cluniac monks envisioned angels as endowed with the characteristics of (i) sexual purity, (ii) capacity for an enhanced psalmody, and (iii) reverential silence, and they regarded their monastic life as an ascetic essay for angelic imitation (Bruce, 2007). Observing the Rule of St. Benedict on *taciturnitas*, and twelfth-century Bernard of Cluny's (1726) direction that *traditum est a Patribus nostris* & *praefixum ut perpetuum silentium tencatur* [it was consigned and prescribed by our Fathers to be kept in perpetual silence], they predicated a vow of silence, which was to be particularly observed during the daily Major (from around 20:00 until sunrise) and Minor (from around noon to 15:00). Thus, in order to circumvent the silence imposed by the strict monastic rule, they created a sign language that they taught and employed during silence periods^8^.

Other cultural niches highly valuing silence have also led to the development of complex sign language systems. One such case is the Ottoman Sign Language (Miles, 2000; Richardson, 2017). This is an archetypal case of niche-construction in that the discreteness sought by Sultans—at least since Mehmet II (r. 1451–81)—imposed a court with the presence of “tongueless” (Turkish *dilsiz*, Persian *bizebani*), which could not speak of the secrets of the court to strangers. This led to a community of hearers and deaf communicating with each other in a sign language, which is reported to be able to express anything, and that was employed by the Sultans themselves.

Last, a more recent case is that of Harsnerēn, or the Sign Language of the Armenian Bride, which is a sign-language employed by hearing Armenian (and Georgian) women in order to circumvent the *č‘xoskanut‘iwn* speech-taboo imposed onto them upon their marriage, which could last from 1 year up to several decades (Karbelashvili, 1935; Kekejian, 2021, 2022)^9^. During that period, the woman is forbidden from speaking to different people (which could vary: in some households it was restricted to the set of her in-laws, but in others it encompassed her in-laws, uncles, aunts, and even her husband)^10^. Given its particular patriarchal nature, it is a specific type of alternate sign-language in that beyond of being employed by hearing people, the language is employed in bimodal conversations, where often the addressees (husbands, in-laws, etc.) do not talk back to the č*‘xoskan* women in Harsnerēn, but in Armenian.

### 3.2. *Lingua franca*

A rather different type of cultural factor catalyzing modality-choice concerns language-clash situations. In encounters of human groups not speaking a common language, it is often the case that—iconicity playing a central role—they resort to pantomime and gesticulation for a more effective communication. For instance, it is reported that in the first encounters of Europeans and American Indians they resorted to signs in order to communicate in such a culturally diverse situation (Axtell, 2000). Furthermore, according to one of the first *conquistadores* the American Indians themselves talked to each other with signs when they did not know the language of each other (Núñez Cabeza de Vaca, 1542; see also Watts, 2000). Then, it is well-known the employment of the Plains Indians Sign Language (PISL) by American Indian populations of very different cultures as a *lingua franca*.

PISL is often characterized as a property of nomadic hunter-gatherer populations whereby “[t]hose who do the most traveling and meet the greatest number of people of a different tongue, have the greatest necessity for its use, and when this need dies away for any cause, the sign language falls at once into decay” (Scott, 1898, p. 58)^11^. The linguistic system that emerged from such intercultural contacts crystallized in one single language that has been employed by over 40 different American Indian Nations in a wide area stretching from Saskatchewan and British Columbia to South of Rio Grande. Even if it was born as a *lingua franca*, the language has also been employed for other uses such as scouting, warfare, traditional storytelling, and for certain traditional rituals (see Farnell, 1995; Davis, 2010 and references therein)^12^.

## 4. Environmental factors

Last, I would like to mention the effect of environmental factors in the emergence of alternate sign-languages. As a matter of fact, when the auditory channel is impractical, there is evidence that humans tend to resort to the employment of hand gestures for effective communication.

A famous—albeit severely limited—case is that of the codes of modern-day scuba-divers, which are employed to denote different types of actions, give orders, ask questions, refer to different species of fish, etc. (see e.g., Prosser and Grey, 1990; Recreational Scuba Training Council, 2005; Bevan, 2007). However, this is a very limited “language”, far more restricted than the previous cases that I reviewed.

A more interesting case is that of the Sawmill Sign Languages, developed in the extremely noisy working environments of the industrial sawmills in the Pacific Coast of Canada and the USA (Meissner and Philpott, 1975a,b; Johnson, 1977)^13^. In these factories, the sawing is heavily mechanized and performed by loud machinery; in consequence, the noise generated by the system impedes oral communication. Thus, several sign languages have emerged among the operators, displaying canonical aspects of language design such as duality of patterning, compounding strategies, intransitive and transitive sentences, interrogative clauses, and other hierarchically complex structures that allow for conversations among several individuals at a time around topics not only related to technical aspects of the work, but also about personal issues or simply joking.

## 5. Conclusion

Language-external factors can affect language-design. In particular, I have shown that biological, cultural and environmental factors may bias the choice of modality of a language, which generally has substantive structural consequences that go beyond modality and phonology.

## Author contributions

The author confirms being the sole contributor of this work and has approved it for publication.

## Footnotes

^1^Whether these are truly created *ex nihilo* can be discussed of course, as some scholars have argued that these languages are heavily influenced by the local oral languages (see, e.g., Harrington, 1938).

^2^Agent-based modeling techniques have been used to study sign language persistence in populations with a degree of inheritable deafness, showing that factors such as the proportion of deafness in the population, the proportion of hearing carriers of a deaf allele, the population size, the assortative marriage for deafness, and the method of sign language transmission (vertical, horizontal, oblique and grandparental) can have a substantive effect in sign language persitence in the population. See Mudd et al. (2020a,b).

^3^They do not display the complexities of “natural” tactile sign languages, but some “professional” tactile sign languages—restricted to specific usages—are also reported in the literature (e.g., Musa and Schwere, 2018).

^4^The speech taboo period can vary substantively; typically it lasts from some weeks up to a year, but Spencer and Gillen (1904, p. 526) also reported that “[T]here is a very old woman in the camp at Tennant Creek who has not spoken for more than twenty-five years, and who will probably, before very long, pass to her grave without ever uttering another word.”

^5^In particular, (Rose, 1992, p. 135–136) notes that among the Yarralin (Northern Territory) “When a woman's husband dies she immediately acquires the dangerous status of being married to a dead man. She does not speak with words but rather with hand signs because her dead husband might hear her voice and want to return.”

^6^The taboo may be more general, as observed by Taplin (1879, p. 23) among the Maraura or Marrawarra (South Australia) “When anyone dies, named after anything, the name of that thing is at once changed. For instance, the name for water was changed nine times in about five years on account of the death of eight men who bore the name of water. The reason is, the name of the departed is never mentioned from a superstitious notion that the spirit of the departed could immediately appear if mentioned in any way.”

^7^In some populations the ban extends to anyone avoiding uttering words that resemble the deceased one's name in front of the widow.

^8^Not only in Cluny; the prescription of silence and the employment of sign language was also adopted by many of the Catholic orders that were influenced by the Cluniac reforms [*i.e.*, the Cistercians (Barakat, 1975), the Order of Sempringham (Graham, 1901; Laughton, 1913), the Christ Church cathedral of Canterbury (Banham, 1991), the *Congregatio Victorina* (Martène, 1764), the Bridgettines (Aungier, 1840), the Trappists (Hutt, 1968), etc.] Ward (1928) also proposed the use of such sign languages among the freemasons and other secret societies.

^9^Armenian women were expected to be modest and virtuous, and silence was held to be an essential ingredient of modesty and respect towards those around them.

^10^According Kekejian (2022), č*‘xoskanut‘iwn* and Harsnerēn are still alive in the Armenian provinces of Tavuš and Gełark'unik'.

^11^Webb (2022 [1931], p. 68) observes that “Practically all students of the sign language are agreed that it originated in the necessity of intertribal communication among a roving nomadic race”, also Mooney (1912, p. 567) notes that “It seems never to have extended west of the [Rocky] mountains, excepting among the Nez Percés and other tribes accustomed to make periodic hunting excursions into the plains, nor to have attained any high development among the sedentary tribes in the eastern timber region.[...]”

^12^See also Tree (2009) for a Mesoamerican instance of sign language use as a lingua franca (which is also employed as a ritual language).

^13^See also Harrison (2014) for an initial study of the signs of a different factory setting.
